# A pilot study to develop assessment tools for Group A Streptococcus surveillance studies

**DOI:** 10.7717/peerj.14945

**Published:** 2023-03-14

**Authors:** Janessa Pickering, Claudia Sampson, Marianne Mullane, Meru Sheel, Dylan D. Barth, Mary Lane, Roz Walker, David Atkinson, Jonathan R. Carapetis, Asha C. Bowen

**Affiliations:** 1 Wesfarmers Centre for Vaccines and Infectious Diseases, Telethon Kids Institute, University of Western Australia, Nedlands, Australia., Perth, Australia; 2 School of Medicine, University of Western Australia, Crawley, Perth, Australia; 3 Sydney School of Public Health, Faculty of Medicine and Health, The University of Sydney, Sydney, Australia; 4 National Centre for Epidemiology and Population Health, ANU College of Health and Medicine, The Australian National University, Acton, ACT, Canberra, Australia; 5 Faculty of Health and Medical Sciences, The University of Western Australia, Perth, Perth, Western Australia; 6 Broome Regional Aboriginal Medical Service, Broome, Australia; 7 School of Population and Global Health, University of Western Australia, Perth, Australia; 8 Ngank Yira Institute for Change, Murdoch University, Perth, Australia; 9 Department of Infectious Diseases, Perth Children’s Hospital, Nedlands, Perth, Australia; 10 Menzies School of Health Research, Charles Darwin University, Darwin, Australia

**Keywords:** Rheumatic fever (RF), Group A Streptococcus (GAS), GAS pharyngitis, GAS impetigo, GAS skin sores

## Abstract

**Introduction:**

Group A *Streptococcus* (GAS) causes pharyngitis (sore throat) and impetigo (skin sores) GAS pharyngitis triggers rheumatic fever (RF) with epidemiological evidence supporting that GAS impetigo may also trigger RF in Australian Aboriginal children. Understanding the concurrent burden of these superficial GAS infections is critical to RF prevention. This pilot study aimed to trial tools for concurrent surveillance of sore throats and skins sore for contemporary studies of RF pathogenesis including development of a sore throat checklist for Aboriginal families and pharynx photography.

**Methods:**

Yarning circle conversations and semi-structured interviews were performed with Aboriginal caregivers and used to develop the language and composition of a sore throat checklist. The sore throat story checklist was combined with established methods of GAS pharyngitis and impetigo surveillance (examination, bacteriological culture, rapid antigen detection and serological tests) and new technologies (photography) and used for a pilot cross-sectional surveillance study of Aboriginal children attending their health clinic for a routine appointment. Feasibility, acceptability, and study costs were compiled.

**Results:**

Ten Aboriginal caregivers participated in the sore-throat yarning circles; a checklist was derived from predominant symptoms and their common descriptors. Over two days, 21 Aboriginal children were approached for the pilot surveillance study, of whom 17 were recruited; median age was 9 years [IQR 5.5–13.5], 65% were female. One child declined throat swabbing and three declined finger pricks; all other surveillance elements were completed by each child indicating high acceptability of surveillance assessments. Mean time for screening assessment was 19 minutes per child. Transport of clinical specimens enabled gold standard microbiological and serological testing for GAS. Retrospective examination of sore throat photography concorded with assessments performed on the day.

**Conclusion:**

Yarning circle conversations were effective in deriving culturally appropriate sore throat questionnaires for GAS pharyngitis surveillance. New and established tools were feasible, practical and acceptable to participants and enable surveillance to determine the burden of superficial GAS infections in communities at high risk of RF. Surveillance of GAS pharyngitis and impetgio in remote Australia informs primary RF prevention with potential global translation.

## Introduction

Rheumatic heart disease (RHD), is the most serious sequelae of rheumatic fever (RF), and the predominant cause of acquired heart disease in children in low resource settings worldwide (Pandey, Batzloff & Good, 2012; Ralph & Carapetis, 2013; Carapetis, Currie & Mathews, 2000; Richmond & Harris, 1998). The burden of RF and RHD experienced by Australia’s Aboriginal population is high (Sims Sanyahumbi et al., 2016). Recent data estimates the combined prevalence of RHD amongst four Australian states (Queensland, Western Australia, South Australia, Northern Territory) at the end of 2020 to be 61.3 per 100,000; a prevalence of 936.1 per 100,000 is recorded for the Northern Territory (AIHW, 2022). Improved understanding of GAS pathogenesis and development of RF is critical for targeted primary prevention strategies including vaccines.

RF is an autoimmune-mediated response that classically follows two to three weeks after group A *Streptococcus* (GAS) pharyngitis (Wannamaker, 1973; McDonald, Currie & Carapetis, 2004). Primary prevention strategies (*e.g.*, early diagnosis and treatment of GAS pharyngitis) have been based on the concept that RF is only caused by GAS pharyngitis (RHD Australia (ARF/RHD writing group) (2012)). Amongst Australian Aboriginal populations, rates of RF and RHD are high (Sims Sanyahumbi et al., 2016), but pharyngitis rates have been inconsistent and reported as either common or uncommon in remote living Australian Aboriginal children (McDonald, Currie & Carapetis, 2004; Noonan et al., 2013; McDonald et al., 2006b). The prevalence of GAS throat carriage is rarely detected or reported (McDonald, Currie & Carapetis, 2004; Carapetis et al., 1997; Van Buynder et al., 1992). In contrast, GAS impetigo is endemic (Yeoh et al., 2017).

For decades, it has been considered plausible that in remote Australia, RF may follow impetigo (RHD Australia (ARF/RHD writing group) (2012); McDonald et al., 2006b), however demonstrating that a significant proportion of RF follows impetigo rather than pharyngitis in this setting is difficult. In New Zealand (where high RF burden exists), contemporary efforts to prevent RF are considering the contribution of other GAS infections, including impetigo (O’Sullivan et al., 2017; Oliver et al., 2021; Thomas et al., 2021). If GAS impetigo also contributes to RF, a greater understanding of the concurrent burden and transmission of GAS impetigo and pharyngitis in endemic, tropical and resource-limited settings is required to effectively employ primary prevention strategies. The contribution of impetigo has been coined the proverbial ‘missing piece’ of the RF narrative. This knowledge gap is narrowing, as highlighted by a recent preprint report of widespread transmission between impetigo and throat carriage in an Australian longitudinal household cohort study (Lacey et al., 2022). The ability to measure the concurrent burden of GAS impetigo and pharyngitis relies upon feasible clinical assessment methodologies for surveillance in remote settings, where the burden of RF is highest.

We hypothesized that one possible reason that sore throats are not commonly reported to clinic by children or their care givers in remote settings was that there may be different words and stories used to describe sore throats in the dialogue between caregivers and health care workers. Consideration of Australian Aboriginal and Torres Strait Islander language in sore throat surveillance has not previously occurred but is likely to underpin precise data collection of associated symptoms. Indeed, there is a scarcity of data available (globally) describing patient experiences of sore throat, with clinical examinations predominating sore throat surveillance.

To address this, our study aimed to develop a sore throat checklist to be used in GAS pharyngitis surveillance from ‘yarning’ - an Aboriginal and Torres Strait Islander Peoples way of learning and communicating in groups that has been adapted for qualitative research (Bessarab & Ng’andu, 2010). We further aimed to integrate the developed sore throat checklist and pharynx photography with methodologies previously designed for remote settings (including sample transport and impetigo imaging) (Bowen et al., 2014b; Bowen et al., 2014a). A pilot study cohort of children was then recruited to collect information on the cost, time and practicality of new and existing tools to determine the concurrent burden of GAS impetigo and pharyngitis in Aboriginal children at high risk of RF.

## Materials & Methods

### Ethics and study setting

This study took place at an Aboriginal Community Controlled Health Organization primary health care clinic in Broome, the largest town of the Kimberley, Western Australia (WA) in September and October 2017. Local community and key stakeholders were consulted over the preceding 6 months. This included consultation with clinic staff to ensure the study was a shared priority and could be conducted concurrently with clinical activities. Letters of support were obtained from the clinic and the Kimberley Aboriginal Health Planning Forum Research Subcommittee (KAHPF, 2017-008). Ethics approval was granted by the Western Australian Aboriginal Health Ethics Committee (Approval 782) and University of Western Australia Human Research Ethics Office (RA/4/1/9206).

### Yarning circle to develop culturally informed sore throat checklist

**Figure 1:**
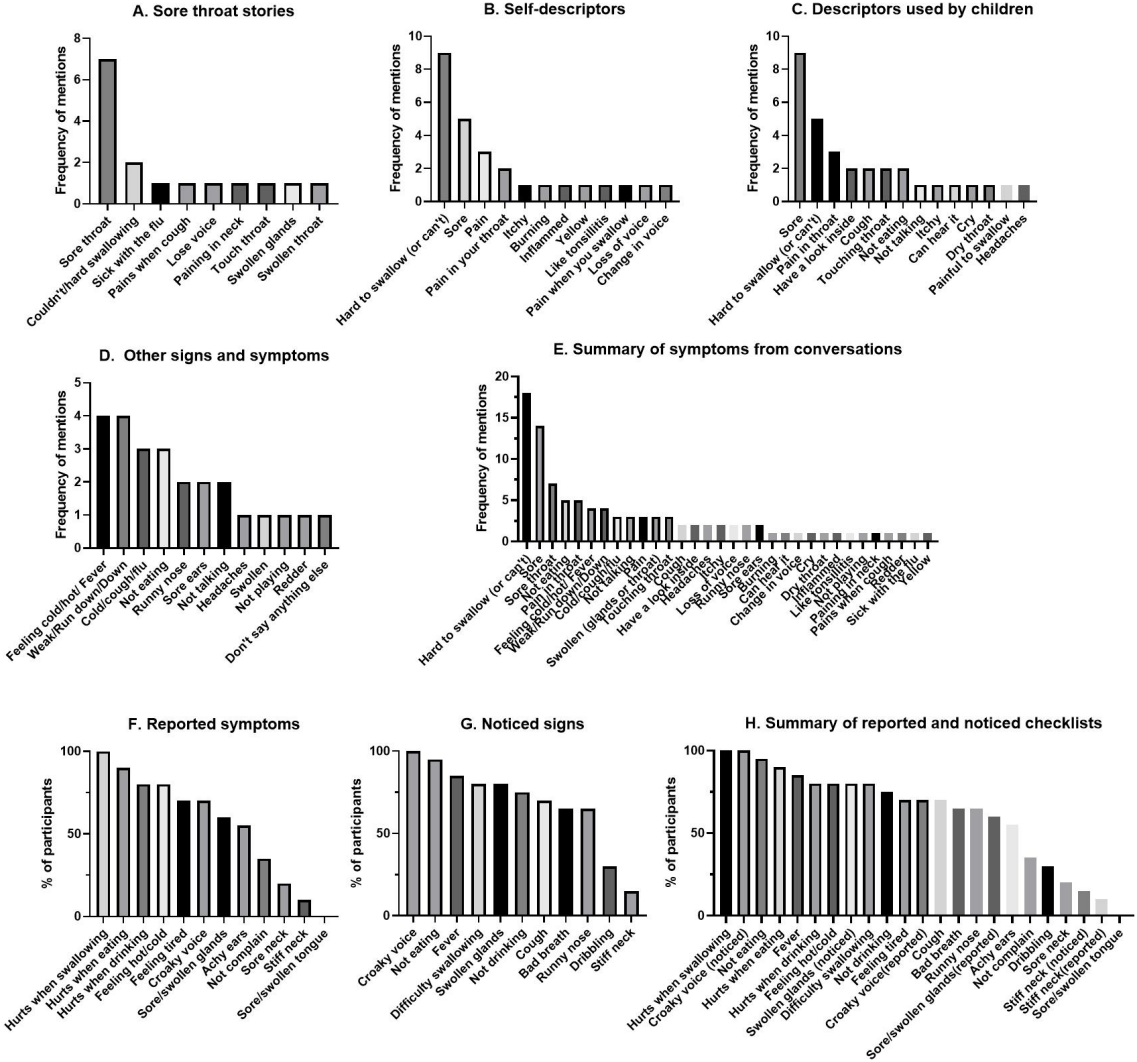
Comprehensive results from yarning circle showing frequency of different symptoms and signs reported to describe a sore throat. From the most frequent reported signs and symptoms, the sore throat checklist was compiled (Table 2).

Over five days in September and October 2017, caregivers of and health care workers for Aboriginal children were invited to participate in a yarning circle to ascertain the preferred words and stories used to describe sore throats in children living in the Broome region of Western Australia. Study staff obtained informed consent and performed semi-structured interviews with open ended questions and symptom conversation triggers (Table 1). A qualitative researcher with decades of experience using the methodology of yarning (Bessarab & Ng’andu, 2010) with Aboriginal families informed the semi-structured interview guide and trained study staff. The yarning circle was led by a non-Aboriginal researcher accompanied by an Aboriginal health care worker who had recruited participants for the research. Yarnings were recorded with permission and transcribed verbatim. Recruitment of participants ceased when data saturation was achieved. Each transcript was reviewed individually; the breadth of sore throat symptoms was collated from all interviews and grouped across eight contextual categories (sore throat stories, self-description, local words, child description, awareness, spontaneously mentioned symptom, checklist reported symptom, checklist noticed signs). The most frequently described sore throat symptoms (Figs. 1A–1H) informed the finalized list of questions for the ‘sore throat story checklist’ for use in the cross-sectional pilot surveillance study (Table 2).

**Table 1:** Open-ended (A) and structured (B) trigger questions used to support yarning about sore throats.

***A: Open-ended sore throat yarning questions***	
A1. Have any of the children you’ve ever cared for had a sore throat? Can you tell us a story?	
A2. How would you describe a sore throat?	
A3. Are there any local words you would use for sore throat?	
A4. How do you know if your child has a sore throat?	
A5. What words would they use?	
A6. Do you think a child you’ve cared for could have had a sore throat without you knowing?	
A7. Sometimes when kids have sore throats, they’ve got other stuff going on at the same time - what else do they tell you about/you notice?	
***B: Symptom checklists used to prompt sore throat conversations***	
B1: When your child has had a sore throat, **did they ever tell you** about other things going on at the same time? 1. Feeling tired 2. Hurts when eating 3. Hurts when drinking 4. Hurts when swallowing 5. Having a sore or swollen tongue 6. Having a sore neck 7. Having a stiff neck 8. Feeling hot or cold 9. Croaky voice 10. Sore or swollen glands when they are touched (demonstrate on the neck) 11. Achy ears 12. No, they never complain	B2: When your child has a sore throat **do you notice** any of the following things about them **[check list, to trigger conversation]:** 1. Bad breath 2. Not eating 3. Not drinking 4. Difficulty swallowing 5. Dribbling 6. They have a stiff neck 7. They have a fever 8. They have a cough 9. They have a runny nose 10. They have swollen glands in the neck 11. Croaky voice

**Table 2:** Sore throat symptom checklist.

	Does the participant have any of the following symptoms **today**? Participants could respond yes, no or unknown.
1	Throat pain or a sore throat
2	Hard to swallow
3	Not eating as much
4	Not drinking as much
5	Croaky voice
6	Fever or feeling hot or cold

### Study pilot and participants

The cross-sectional pilot surveillance study took place in the same clinic setting as the yarning circles and was performed over two days in October 2017. Caregivers of participants were approached in the waiting room. The primary purpose for their attendance was not recorded but could be to access healthcare for children or adults. Caregivers of Aboriginal children aged 3–15 years provided written informed consent for their child/children; children ≥7 years provided written assent. Children with immune deficiency and known diagnosis of RHD and who were receiving secondary prophylaxis with benzathine penicillin G (BPG) were excluded. A sample of 15 participants was considered achievable to recruit and assess over 2 days. With their caregiver present, children were interviewed and assessed by a student doctor and a pediatrician. A clinical reference form that collected data was used for data collection. Demographic information (age, Aboriginal status, gender, mean number of people and bedrooms in household, eligibility, and school attendance) were recorded. To assess for GAS impetigo and pharyngitis, research study staff performed: (1) a checklist of questions to ascertain current sore throat and skin symptoms, (2) clinical assessments, (3) photographs, (4) bacteriological tests, and (5) serological tests, which are detailed (Table 3). All children were seen on the same day by their usual care clinician and offered treatment, where indicated, for pharyngitis or impetigo. Data were recorded in the web-based Research Electronic Data Capture (REDCap) platform (Harris et al., 2009) (Vanderbilt, Nashville, USA) during the assessment.

**Table 3:** Overview of assessment tools and methodologies used for simultaneous assessment of GAS impetigo and pharyngitis in children.

	History	Clinical Examination	Photographs	Bacteriological tests	Serological tests
GAS Pharyngitis (throat infection)	Checklist of symptoms in children^*^Table 2	Modified Centor Criteria (Harris et al., 2009) + Tonsillar Hypertrophy Grading Scale (McIsaac et al., 2000)	Pharynx^#^	RADT (Brodsky, 1989) + GAS culture (Bowen et al., 2014c)	ASO (blood spot) (Andrews et al., 2009)
GAS Impetigo (skin infection)	Checklist of symptoms (Bessarab & Ng’andu, 2010; Menzies School of Health Research (2009))	Sore type and frequency (Sampson, Mullane & Bowen, 2017)	Skin sores	GAS culture (Bowen et al., 2014c)	ASO (blood spot) (Andrews et al., 2009)

**Notes.**

* Developed from yarning circles with Aboriginal parents, caregivers and health care providers.

# Developed for this study based using a photography protocol.

### Symptom data collection and examination

Children were screened for pharyngitis using the checklist of six questions (Table 2) that were developed from the sore throat yarning sessions. The Modified Centor Criteria (McIsaac et al., 2000), a validated tool for throat examination, was used to determine the risk of GAS pharyngitis. Tonsillar size was assessed by the research study clinician using the Brodsky scale (Brodsky, 1989), a standardized and reproducible measurement. The skin symptom checklist was previously validated in a surveillance program and clinical trial and used without change (Bowen et al., 2014c; Andrews et al., 2009). Examination of the visible skin recorded purulent, crusted and flat/dry sores on a body map using a standardized tool (Recognition and Treatment of Skin Infections protocol) (Menzies School of Health Research, 2009). When a child indicated a skin sore under their clothes, these sores were examined and documented.

### Photography

An iPhone 7 Plus (Apple Inc., Cupertino, USA) mobile device was used to conduct all digital photography of the pharynx and skin. Approaches to pharynx photography were developed into a standard operating procedure with the assistance of the Perth Children’s Hospital (PCH) otolaryngologists and hospital photographer (Sampson, Mullane & Bowen, 2017). Photographs of the pharynx and skin used standardized equipment and camera settings, participant positioning and photography technique. Photographs were then uploaded to REDCap for objective grading of the tonsils using the Brodsky Grading Scale (Brodsky, 1989) by two independent pediatricians. This independent assessment aimed to provide data on the feasibility and accuracy of using photographs to help assess sore throats in other clinical applications, such as in remote and telehealth contexts. For each participant, the most severe skin sore was photographed using an iPhone adapted version of the photographic protocol described by Bowen et al. (2014c)

### Bacteriological tests

Throats were swabbed simultaneously for GAS Rapid Antigen Detection Testing (RADT) (Stewart et al., 2014) and culture by holding two sterile swab sticks together while passing them over the tonsils and avoiding the tongue. The RADT (Alere BinaxNow GAS, Abbot, Chicago, USA) was performed immediately according to manufacturer’s instructions. The most severe skin sore was swabbed as previously described. (Bowen et al., 2014c; Bowen et al., 2014d) Throat and skin swabs were placed in vials of skim milk tryptone glucose glycogen broth (STGGB, PathWest Media, Perth, Australia), and kept at 4–8 °C until culture at the PathWest diagnostic laboratory, Broome, WA. GAS were identified (Andrews et al., 2009) from 10 µL of STGGB streaked on horse blood agar plates and incubated for 24 h at 35–37 °C. Suspected isolates were confirmed with a latex streptococcal grouping kit (Oxoid, Basingstoke, United Kingdom). The common skin pathogen, *Staphylococcus aureus,* was also identified from skin swabs with positive slide (Oxoid) and tube coagulase results, with methicillin susceptibility confirmed by the cefoxitin disc diffusion method (FOX 30, Oxoid, United Kingdom) (Bowen et al., 2013).

### Serological tests

Blood was collected from a single fingerprick sample; blood droplets squeezed onto Whatman 903 filter paper (Interpath, Perth, Western Australia) to fill all five circles. The resulting dried blood spot (DBS) samples were air dried at room temperature for 24 h and then stored with desiccant at 4–8 °C for transportation to Perth, WA. Two blood spot circles were independently processed per card to detect anti-streptolysin O (ASO) antibodies at PathWest Laboratories (Perth, Australia) on a nephelometer as previously described (Joseph et al., 2017).

### Feasibility, acceptability, and budgeting for scaled surveillance

Throughout the study, participants and caregivers were asked about their comfort, willingness to continue and whether they had any questions. Feedback from participants was sought to determine the acceptability of the consenting process and their experience with each aspect of data collection in field notes by study staff about the questionnaires, clinical checks, throat skin and blood sampling and photography) during their participation. Study notes of participant feedback and the ease or difficulty of performing each assessment tool were captured by study staff and compiled as raw data at the end of the pilot. To inform future scaling of throat and skin surveillance, study staff calculated the time taken to perform each aspect of the assessment, staffing and consumable costs. The total cost for the surveillance pilot was calculated, and an average cost per child determined.

### Data analysis

Statistical analysis of descriptive and inferential statistics used GraphPad Prism 9.1.2 (GraphPad Software Inc, San Diego, CA, USA).

## Results

### Sore throat yarning reveals key sore throat descriptors

Sore throat yarning achieved data saturation with ten Aboriginal participants. All were mothers (five aged 25–39 years, five aged 40–59 years) that had additional caring roles to other children including as grandmothers (*n* = 5), aunts (*n* = 10), and carers or guardians (*n* = 7). Stories about caring for children with sore throats identified nine symptoms, whereas self-descriptors identified 12 symptoms (Figs. 1A, 1B). ‘Sore throat,’ and ‘hard to swallow’ were the predominant symptoms. No local words were used by participants to describe a sore throat. Additional descriptors were identified by ‘those used by children’ (Fig. 1C) and included ‘cough’, ‘touching throat’, ‘not eating’, and ‘not talking’, however soreness and hard to swallow remained the predominant descriptors. Other signs and symptoms revealed less commonly reported symptoms (Fig. 1D) with feeling hot/cold and weak/run down predominating. Thirty-two symptoms were recorded from conversational prompts (Fig. 1E). One participant identified there was a possibility of a child not reporting a sore throat “*so that [child] could keep playing*.” The remaining participants reported high awareness for sore throat illness in children they cared for. Pre-defined checklist prompts for ‘reported’ and ‘noticed’ symptoms (Figs. 1F and 1G) identified 22 symptoms, including additional descriptors for the sore throat story (croaky voice, achy ears, bad breath). Croaky voice was an addition to the ‘predominant descriptors’ that were first identified in conversations (hurts when swallowing, not eating/drinking, sore, feeling hot/cold). Six predominant descriptors were chosen for the sore throat checklist (Table 2). Despite being recorded as prevalently in some instances, the descriptors, ‘tired,’ ‘weak’ and ‘run-down’ were not considered specific for sore throat and therefore excluded from the developed checklist.

### Symptom data collection and examination

Children were assessed in family groups of between 1–5 children at a time. Of the 21 children approached, 17 were eligible and recruited (mean age 9 years [interquartile range (IQR) 5.5–13.5], 65% female). Of the 17 children, three declined the finger-prick test and one the throat swab. All other elements were completed by all children.

Seven participants answered affirmatively to one or more of the sore throat symptoms questions (Table 2), in two of whom the sole symptom ‘feeling hot or cold’, five reported ‘throat pain’, four ‘hard to swallow’, two ‘not eating’, two ‘croaky voice’ and six ‘feeling hot or cold’. ‘Not drinking’ was the only sore throat descriptor not affirmatively reported in this pilot. Nine (53%) participants had a Modified Centor Criteria score >1 on examination, with 3/17 declared to have had prior tonsillectomy. Clinical examination identified two children with a Modified Centor score of 2 (due to age and absence of cough), however as these children were asymptomatic, this score was not clinically relevant. Consequently, appropriate clinical examination did not identify sore throats in addition to those reported by children using the sore throat story.

Skin infection symptoms were reported by 5/17 (29%) participants with a reported duration of <1 day (*n* = 1), 1–3 days (*n* = 2), 3–5 days (*n* = 1) and >5 days (*n* = 1). Four reported these sores were itchy, whilst three reported that they were painful/uncomfortable. On clinical examination, each of these children were recognized to have current impetigo lesions (crusted and purulent). Clinical examination identified an additional ten children with skin sores. Of the 15 total children with skin sores, 11 presented with a combination of ‘purulent and crusted’ or ‘purulent and flat/dry’ impetigo. Two children presented with scabies. The total number of sores ranged from 0–42 (mean 7.5, median three [IQR 1.5−9.5]).

Three children reported sore throat and skin sores concurrently, four a sore throat only and two skin sores only. Clinical examination identified concurrent sore throat and skin sores in seven children, a further two children with skin sores only, and no children with sore throats only.

### Photography

**Figure 2:**
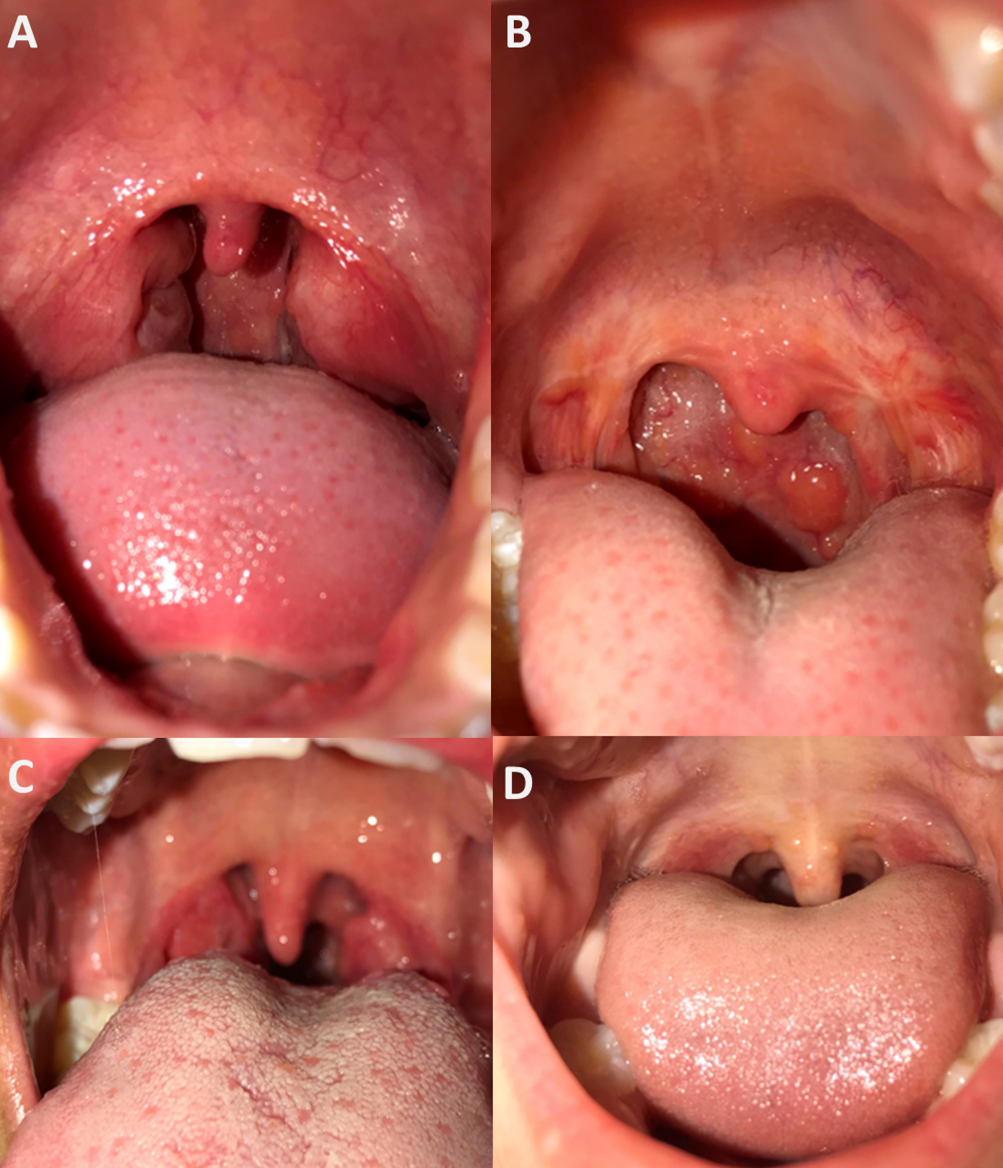
Examples of pharynx photography. Assessment by independent clinicians determined images (A) and (B) to be of good image quality and high pharynx visibility, image (C) was considered poor image quality and high tonsil visibility and image (D) to be of good image quality and partial tonsil visibility.

All participants consented to throat photography (Fig. 2). Two independent clinicians assigned Brodsky grading scales for all pharynx images that were deemed good quality (15/17). Study notes recorded the two instances of difficultly with photography were due to a reluctance for one participant to open their mouth wide for photography, and a small oral cavity obscuring the pharynx in another. On clinical assessment, the median Brodsky grading score was two, consistent with the median Brodsky grading score from photographs of 1.5. The clinically assigned score on the day and that assigned *via* photograph analysis was only equivalent on five of 15 occasions (grade 0 = twice, grade 1 twice, grade 3 once). Of those that differed, nine were a single score apart, and one was two scores apart. Photography captured photo evidence of tonsillectomy in two children, with one also having evidence of pharyngitis.

### Bacteriological and serological testing

All RADTs were negative (*n* = 16). Throat swabs from 4 participants identified scanty GAS, scored as 1+ out of a possible 4+ for each of the plates. All four children who had GAS cultured from throat swabs were referred for treatment with intramuscular benzyl benzathine penicillin as a conservative approach to prevent RF. Of these four, only one presented with sore throat symptoms (throat pain, croaky voice and fever) for which she had attend clinic for care on the same day. Four additional participants cultured group C *Streptococcus* from throat swabs of which two presented with sore throat symptoms –none were referred for treatment. Fifteen participants had skin sores swabbed. Of the 15 sore swabs, one cultured GAS and this same participant had GAS in the throat with symptomatic sore throat presentation. Three participants cultured *S. aureus* from skin swabs of which one was identified as methicillin resistant (MRSA). GAS and *S. aureus* co-infection was found in 1/15 sores. Of 14 participants consenting to DBS, the median anti-Streptolysin O titer (ASOT) was 306 IU/mL ([IQR] 220.3–776.8). Most ASOT values (12/14) exceeded the upper limit of normal (<200IU/mL) (Steer et al., 2009).

### Feasibility and acceptability

Acceptability and engagement were inferred from study notes, the positive responses, high but not universal consent rates, engagement in activities and low rates of refusal to any aspect of the pilot study. The participating children were very engaged in the assessments, had high uptake for the more invasive procedures *e.g.*, throat swab (16/17) and finger prick (14/17) and enjoyed assisting with the RADT processing as they were able to understand the science of why their throats were being swabbed. Participants and their families asked appropriate knowledge seeking questions throughout the interaction. Although three children did not consent to the finger-prick DBS, this was reported by the carer to coincide with historical resistance to blood draws.

**Figure 3:**
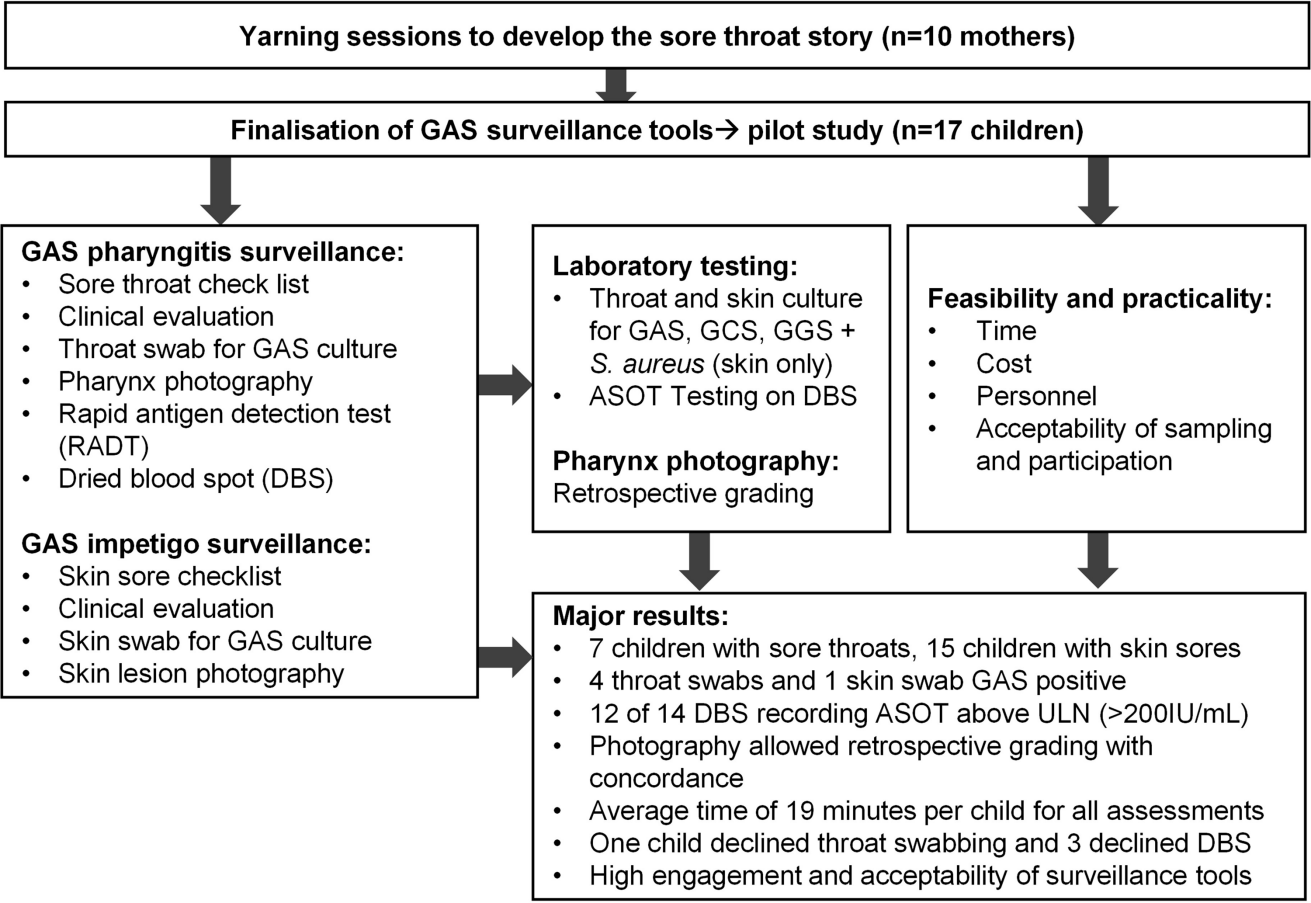
Flow diagram of steps in study and major results. ASOT = anti-streptolysin O testing, ULN = Upper limit of normal.

The total mean time for screening was 19 min per child (standard deviation [SD] ±5.6 min), with a mean of 23 min on day one and 16 min on day two. The fastest assessment in participant 16 of 17 was 11 min, with two study staff collecting data. It was noted that confidence with the surveillance activities improved by the research team with progressively shorter assessment times. Surveillance costs per child was estimated to be $89.63 AUD. Other estimated costs of the study, including once-off costs were recorded (Table 4). A flow diagram indicating steps in study and major findings is presented (Fig. 3).

## Discussion

This pilot study confirmed the first, culturally appropriate sore throat story for Australian Aboriginal children and that skin sore and sore throat assessments to improve the understanding of RF pathogenesis and prevention, can be conducted concurrently. The pilot was feasible, affordable, and accepted by participants and their families.

In this study, ‘Yarning About Sore Throats’ enabled collaborative development of a culturally informed, sore throat check list. The inclusion of ten Aboriginal carers in yarning sessions, the data saturation achieved and congruence amongst sore throat descriptors strongly clarified the six most used words (of 32 used in total) for selection in the finalized checklist. The use of this tool was subsequently tested in the pilot study, indicating the appropriateness for sore throat surveillance in Aboriginal families living in Broome, a population with higher risk of RF. The yarning process involved a combination of open answered questions and checklist responses enabling free dialogue but also a systematic approach to ensuring each potential descriptor was discussed with each mother. The inclusion of an Aboriginal co-researcher in the yarning strengthened the process and contributed to open dialogue amongst participants.

**Table 4:** Financial cost of pilot study, including cost estimate per child and set-up costs in AUD.

	**Details**	**Cost estimate**
Consumables	Including skin and throat swabs, STGGB vials, RADTs, Throat Scope tongue depressors, DBS collection apparatus, and an allowance for other consumables *i.e.*, Band-Aids, cotton wool buds, gloves^*^.	$15.71 / child
Staffing cost	Staff time to complete throat and skin assessment: Estimated ∼15 min per child.	$12 / child
	Staff time to complete lab processing and follow up: Mean 1.29 h per child.	$61.92 / child
Total estimated cost of study (not including one-time costs)		$89.63 / child
One-time costs	REDCap Database production.	$2625
	Equipment including iPhone 7 Plus, laptop and covers/cases.	$3200

**Notes.**

Staffing costs estimated using remote nurse salary of $48 per hour. These prices are from 2018/19 and do not account for economic inflation.

* Excludes long-term isolate storage costs.

We did not identify any local words used to describe sore throats. However, to our knowledge, this is the first time Aboriginal families have been involved in development of a sore throat story for clinical care and research in remote Australia. Similar research with other Aboriginal nations in northern Australia is needed to validate this approach as different language and cultural groups may have different findings. Inclusion of the sore throat checklist enhanced the approach for assessment of GAS pharyngitis, and there was good concordance between child reported sore throat symptoms and evidence of sore throat on clinical examination. Further work to derive knowledge from the sore-throat checklist data is currently underway (Barth et al., 2022b), and will be important for enriching culturally informed surveillance.

Earlier attempts to understand the concurrent burden of superficial GAS infections focused on households at very high risk of RF due to a family member already receiving a diagnosis of RHD (McDonald et al., 2006a; McDonald et al., 2007). These studies confirmed the very high burden of skin infections but were not consistent on pharyngitis rates. Our study, more than a decade later, has further developed surveillance tools for remote research to understand the concurrent burden of GAS impetigo and pharyngitis more conclusively. We chose a clinic setting for the pilot study for practical reasons including strong engagement with the clinic and staff, and access to treatment for children with confirmed infection (if necessary). A limitation is that we did not collect data on the primary purpose for family attendance at the clinic and were therefore unable to compare routine clinical care to our Strep A surveillance methods. Future studies could address this, but should also progress beyond the clinic to survey the community level burden of superficial GAS infections to inform prevention (Yeoh et al., 2017).

This pilot tested the surveillance tools required for co-examination of impetigo and pharyngitis. Our pilot study has generated new hypotheses for further study. Whilst sore throat attendance at remote primary health care clinics have been low (Hendrickx et al., 2018); 7/17 (41%) children in this study, using the culturally appropriate sore throat check list, reported a sore throat. However, only 1/7 children with a sore throat had GAS cultured from throat swabs and the RADT was negative for all. There is limited available data on the performance characteristics of RADT in remote Australia, however these antigen-based tests are likely to be surpassed by newer molecular nucleic acid detection tests which may also provide data needed data on GAS carriage and the development of RF (Ralph et al., 2019; Pickering, Barth & Bowen, 2020). Molecular point of care testing (POCT) for GAS detection may improve this detection considerably (Pickering, Barth & Bowen, 2020; Gunnarsson et al., 2021; Barth et al., 2022a). The remaining 3/17 children with GAS detected met the definition of GAS carriage (De Muri & Wald, 2014) (lacking sore throat symptoms but with a positive GAS culture result). Two of these children provided blood samples with anti-streptolysin O (ASO) titers >300IU/ml but also had concurrent skin sores making causation difficult to assess. Despite a general consensus that pharyngeal GAS carriage does not lead to RF, (De Muri & Wald, 2014) whether or not referral for treatment for GAS detection is warranted in an endemic, high-risk population such as that found in Aboriginal communities of northern Australia is a question that remains unanswered.

An analysis of ten studies of children from remote Indigenous communities of northern Australia estimated a median prevalence of purulent or crusted impetigo (44.5%). (Bowen et al., 2015) In our study with small sample size, we report a higher prevalence (11/17 (65%)); this is more consistent with other earlier studies from Western Australia (Lehmann et al., 2003). GAS was cultured from only 1 purulent lesion, which is lower than expected using the same microbiological sampling conditions from other studies in northern Australia (Bowen et al., 2014d). Future validation of molecular GAS detection from skin may be helpful in confirming whether GAS impetigo contribute to the development of RF in Australian Aboriginal children in a remote setting.

Compared to pharyngitis, there was less concordance between child reported skin infection and clinical examination. This was potentially due to the first skin sore question requiring a ‘yes’ answer to trigger follow up questions about skin sore duration, itchiness, and pain. We conclude that comprehensive clinical examination of skin remains critical for identifying and naming skin sores with children, their families and in discussion with healthcare providers in the Kimberley. This is important as skin sores affect almost one in two children in this region at any one time (Bowen et al., 2015) which can result in normalization such that they are not reported to/noted by health care providers (Yeoh et al., 2017).

Photography has not previously been utilized in sore throat surveillance anywhere in the world. For this reason, clinical assessment on the day was compared with an independent physician assessment of throat photographs. Overall, clinical and photograph assessments were within 1 Brodsky grade supporting the validity of this method. Further assessment in larger cohorts is required. Our protocol has subsequently been used in a human challenge model for GAS pharyngitis in adults and proved useful (Osowicki et al., 2021). Since the completion of our data collection study in 2017, studies in international populations have published the utility of photography for low-cost diagnosis of GAS pharyngitis (Askarian, Yoo & Chong, 2019). Newer technologies and post imaging processes may one day approach a high level of diagnostic accuracy but are currently outperformed by gold standard GAS culture in hand with symptom reporting and clinical examination. Accurate, culturally appropriate surveillance tools will remain critical to the validation of new tools.

The number of children who had had tonsillectomy (*n* = 3) was unexpected considering limited access to Ear, Nose and Throat (ENT) surgery (Australian Institute of Health and Welfare (AIHW), 2011). One of the three children had GAS cultured from their throat swab. As it is unknown whether children without tonsils develop RF (Orvidas, St Sauver & Weaver, 2006), tonsillectomy is unlikely to become an exclusion criteria for future studies; this is important data to include and one advantage to performing pharynx photography.

The time to complete a study visit decreased over the course of the two days as investigator familiarity with the tools increased. Based on this, we estimate that it would take a minimum of 10 min per child for two research staff to complete the assessments as performed in this study. Two study staff were necessary to allow for simultaneous collection and recording of data. This is the first study to report on time taken for surveillance activities and is useful to inform remote health activities where school-based surveillance for trachoma, otitis media and dental health are common (Dossetor et al., 2019). It also informs ethics applications, operational planning and resource allocation for settings such as schools where health surveillance intersects with educational priorities. Once established, this methodology could be integrated into other school-based programs in remote settings.

We estimated the economic cost of this activity to assess whether it is scalable to other settings and larger research studies in Australia. However, we did not arrive at a realistic surveillance estimate due to the small study size making economies of scale not possible. Many costs were dependent on the quantity of items purchased, staff costings and delivery method but it is expected that the cost per child would be reduced in a larger scale study. Informal feedback, behavior, engagement, and ongoing assent suggested that the process was not distressing and appeared enjoyable to many participants. Larger studies should take the potential occurrence of challenges associated with blood draw into consideration with sample size estimates.

## Conclusions

Our study aimed to develop a sore throat checklist to be used in GAS pharyngitis surveillance from ‘yarning’ –and to integrate tools with methodologies previously designed for remote settings. The pilot surveillance study enabled the new sore throat checklist and pharynx photography to be trialed amongst established methods of skin and throat assessments. The advantage of our pilot approach was the ability to test methodologies for concurrent sore throat and skin checks before more widespread application. Overall, we found the comprehensive assessment tools for impetigo and pharyngitis were practical and acceptable to implement for surveillance purposes. The methodologies tested in this pilot study have since led to an integrated protocol for validation in a large school-based surveillance study for GAS impetigo and pharyngitis in Barth et al. (2022b). The learnings from this pilot has informed the need to capture tonsillectomy and focus on more sensitive modern molecular point of care tests for GAS rather than RADTs.

##  Supplemental Information

10.7717/peerj.14945/supp-1 Supplemental Information 1 Raw Data

## Additional Information and Declarations

### Data Availability

The following information was supplied regarding data availability: The raw numeric data are available in the Supplemental Files.
